# Transcriptomic analysis reveals a controlling mechanism for NLRP3 and IL-17A in dextran sulfate sodium (DSS)-induced colitis

**DOI:** 10.1038/s41598-018-33204-5

**Published:** 2018-10-08

**Authors:** Tien-Jen Lin, Shu-Yi Yin, Pei-Wen Hsiao, Ning-Sun Yang, I-Jen Wang

**Affiliations:** 1 Department of Neurosurgery, Wan Fang Hospital, Taipei Medical University, Taipei, ROC Taiwan; 2 Graduate Institute of Injury Prevention and Control, Taipei Medical University, Taipei, ROC Taiwan; 3 Graduate Institute of Sports Science, College of Exercise and Health Sciences, National Taiwan Sport University, Taoyuan City, ROC Taiwan; 4 Agricultural Biotechnology Research Center, Academia Sinica, Taipei, 115-29 ROC Taiwan; 5 Department of Pediatrics, Taipei Hospital, Ministry of Health and Welfare, Taipei, Taiwan; 6 School of Medicine, National Yang-Ming University, Taipei, Taiwan; 7 College of Public Health, China Medical University, Taichung, Taiwan

## Abstract

The incidence of inflammatory bowel disease (IBD) has markedly increased. Our research findings during the past showed that medicinal plant extracts and the derived phytochemical components from *Wedelia chinensis* (WC) can have strong anti-colitis activities. Here, we further identified the key component phytochemicals from active fractions of different WC preparations (WCHA) that are responsible for the protective effect of WCHA in colitis mice. Of the 3 major compounds (wedelolactone, luteolin and apigenin) in this fraction, luteolin had the highest anti-inflammatory effect *in vivo*. Using a next-generation sequencing (NGS) (e.g., RNA-seq) system to analyze the transcriptome of colorectal cells/tissues in mice with dextran sulfate sodium (DSS)-induced colitis with/without phytochemicals treatment, luteolin was found to strongly suppress the DSS-activated IL-17 pathway in colon tissue. In addition, co-treatment with wedelolactone and luteolin had a synergistic effect on the expression level of some IL-17 pathway-related genes. Interestingly, our NGS analyses also indicated that luteolin and wedelolactone can specifically suppress the expression of NLRP3 and NLRP1. Using a 3-dimensional cell co-culture system, we further demonstrated that luteolin could efficiently suppress NLRP3 expression via disruption of IL-17A signaling in inflamed colon tissue, which also indicates the pharmacological potential of luteolin and wedelolactone in treating IBD.

## Introduction

Inflammatory bowel disease (IBD), which occurs in millions of patients worldwide, represents a group of inflammatory disorders of the gastrointestinal tract. Patients who suffer from IBD symptoms also have a high risk of colorectal cancer development^1^. To investigate the cellular or molecular mechanisms of IBD, a dextran sulfate sodium (DSS)-induced colitis mouse model was developed to mimic some inflammatory conditions in human IBD^2^. Many pro-inflammatory molecules abnormally produced by inflamed colon epithelial cells are crucial factors in the pathogenesis of IBD^3^. The secretion of cytokines in the IL-17 family from Th-17 cells can apparently confer specific inflammatory activities in various diseases, including psoriasis, rheumatoid arthritis, multiple sclerosis, asthma and IBD^4,5^. With the increasing number of reports on the roles of Th-17 cells and derived IL-17A secretion, there has been increasing interest in new therapeutic approaches targeting the IL-17A pathway in the treatment of IBD.

An intracellular protein family, namely the nucleotide-binding domain and the leucine-rich repeat-containing (NLR) protein, is a group of receptors that can recognize various patterns of pathogen molecules^6^. Among these NLRs, NLRP3 is known to form an inflammasome complex with ASC (adaptor molecule) and caspase-1, which can further activate IL-1β and IL-18 in different tissue environments^7,8^. Crohn’s disease has been highly associated clinically with the appearance of some single nucleotide polymorphisms (SNPs) in the NLRP3 gene^9,10^. In the DSS-induced colitis model, the NLRP3 inflammasome was also indicated as a key mediator in mouse colon inflammation^11^. These findings suggest that the formation of the NLRP3 inflammasome is another potential target for the development of IBD therapy. Of interest, it has been shown that activation of innate pattern recognition receptors, including toll-like receptors (TLRs) and the inflammasome, is required for generation of IL-17A. Although this pathway leads to the production of IL-17A in models of infectious diseases^12–14^, its relevance in colon inflammation is not clear. Therefore, it is possible that improving our knowledge on the regulatory mechanism of IL-17A signaling for inflammasome activities could provide useful insights into potential therapeutic targets for IBD.

Drugs traditionally used for IBD treatment, such as sulfasalazine, corticosteroids and 5-amino salicylic acid (5-ASA), are anti-inflammatory agents^15^. However, these drugs also have some cytotoxic side effects on the body, such as Cushing’s syndrome and headache induced by long-term administration of corticosteroids^16^. Therefore, some plant-based natural products containing bioactive phytochemicals have been re-recognized recently as preferentially important sources for developing anti-inflammatory food supplements, medical food or even botanical drugs. Our studies during the last few years found that medicinal plant extracts and the derived phytochemical components from *Wedelia chinensis* (*W*. *chinensis*, WC) can have strong anti-inflammatory activities and exhibit potent anti-colitis activities in mouse models^17,18^, indicating their potential application for treatment of human IBD. Therefore, in this study, we further investigated specific phytochemical fractions (WCHA) and derived single compounds, including luteolin, apigenin and wedelolactone, in WC plants with anti-inflammatory and anti-colitis activities. We found that luteolin and wedelolactone can confer strong and substantial anti-colitis activities, respectively. To further investigate the *in vivo* pharmacological mechanism of luteolin or wedelolactone, next-generation sequencing (e.g., RNA-seq) analysis was used to evaluate the transcriptome activity of colorectal cells/tissues in DSS-induced colitis mice with test phytochemical treatments. The aim of this study was to systematically identify possible hierarchical regulatory role of inflammasome components, including NLRP3 and NLRP1, and the activation of the Th-17 pathway in the luteolin- and wedelolactone-mediated suppression of DSS-induced colitis. Some significant changes in the alternative splicing activity of specific genes in DSS-induced inflamed colon tissues were also detected and the significance discussed.

## Results

### Identification of active phytochemicals in specific WC extracts for suppression of DSS-induced colitis in mice

**Figure 1:**
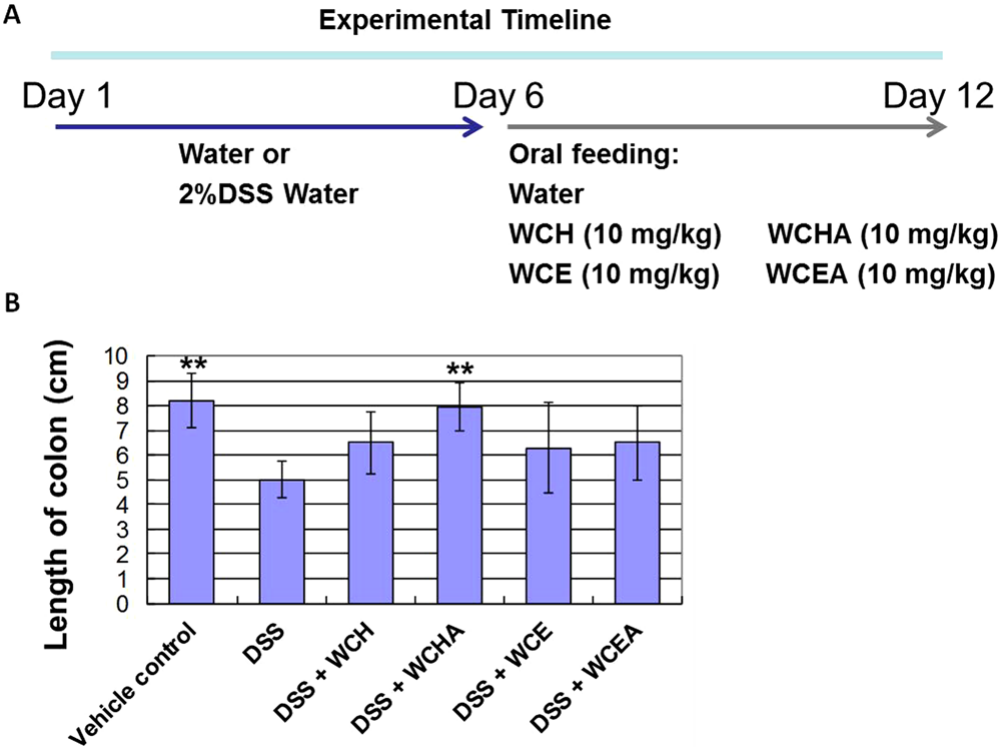
Effects of different *W*. *chinensis* extracts on mice with DSS-induced acute colitis. *W*. *chinensis* extracts were prepared by different extraction methods as described in Materials and Methods. Mice were simultaneously orally fed with 2% (w/v) DSS solution for 6 days, followed by administration of a vehicle (1% Tween 80) or different *W*. *chinensis* extracts (10 mg/kg body weight), respectively, for 6 days. (**A**) Experimental timeline for DSS-induced colitis and oral feeding of extracts and colon length (**B**) are shown. Data are expressed as mean 6 ± SD. (n = 6). WCH: hot water extract of fresh *W*. *chinensis*; WCHA: WCH followed by hydrolysis treatment; WCE: 95% ethanol extracts of *W*. *chinensis*; WCEA: WCE followed by hydrolysis treatment, respectively. **P < 0.01, significant difference compared with the DSS group.

**Figure 2:**
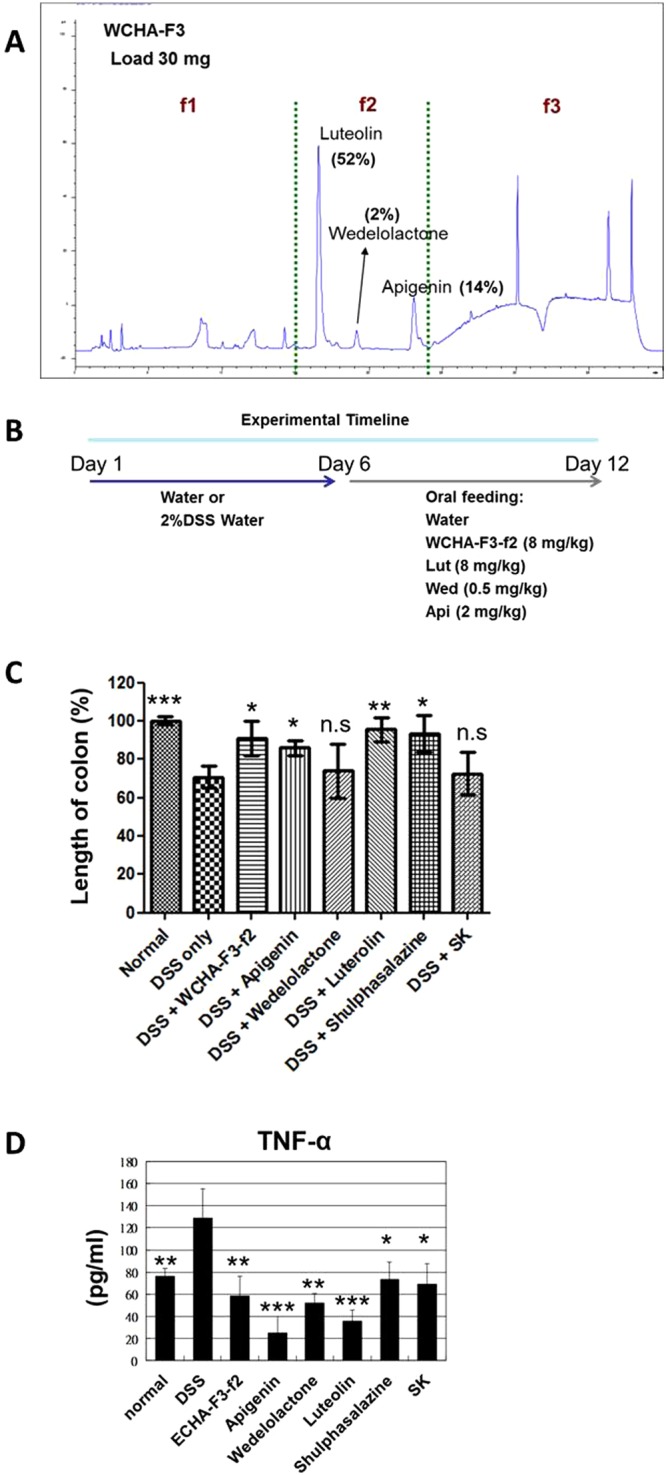
(**A**) Preparative HPLC purification of the fraction WCHA-F3. Identification of active phytochemicals (LAW) in specific *W*. *chinensis* extracts for suppression of DSS-induced colitis. (**A**) The specific WCHA fraction (WCHA-F3) was further fractionated into 3 sub-fractions (WCHA-F3-f1~3) using a C18 column (detection wavelength: 300 nm). The WCHA-F3-f2 contains luteolin (52%), wedelolactone (2%) and apienin (14%). (**B**) Timeline for DSS-induced colitis and oral feeding of phytochemicals. To evaluate bio-active components with anti-colitis activity, colitis in C57 mice was induced by feeding 2% (w/v) DSS solution in drinking water from Day 0 to 6. Test mice then were orally fed WCHA-F3-f2 (8 mg/kg), luteolin (8 mg/kg), wedelolactone (0.5 mg/kg), apigenin (2 mg/kg), sulfasalazine (100 mg/kg) or shikonin (SK; 10 mg/kg) from Day 6 to 12. Changes in colon length (**C**) and TNF-α secretion (**D**), in response to each phytochemical treatment, were expressed as mean ± SD (n = 6). *P < 0.05, **P < 0.005, ***P < 0.001, significant difference compared with the DSS group.

To compare the anti-colitis activity of different WC extracts, test mice were treated orally with 2% DSS in drinking water for 6 days from the beginning of the experiment, followed by oral feeding of either vehicle solution, WCH extract, WCHA, WCE or WCEA (all at 10 mg/kg body weight) for another 6 days (Fig. 1A). Among different types of WC extracts, we found the hot water extract of WC followed by hydrolysis treatment (WCHA) had higher effect than the other extracts (Fig. 1B). Furthermore, we also found that the third fraction of hot water extract of WCHA (WCHA-F3) had higher effect activity than the other WCHA fractions. To evaluate the bio-active components in WCHA-F3 with anti-colitis activity, WCHA-F3 was further fractionated into 3 sub-fractions (WCHA-F3-f1~3), based on FPLC profile (Fig. 2A). Using the same DSS-induced colitis model, test mice were treated orally with DSS for 6 days, followed by the vehicle solution, WCHA-F3-f2 extract (8 mg/kg body weight), luteolin (8 mg/kg body weight), apigenin (2 mg/kg body weight) or wedelolactone (0.5 mg/kg body weight) for 1 week (Fig. 2B). Here, the administered dosages of each phytocompound were designed in consideration of their composition ratio in WCHA-F3-f2. By comparing the length of the colon in test mice in response to each phytochemical (e.g., luteolin, wedelolactone and apigenin) treatment, we found that oral administration of luteolin can very effectively suppress DSS-reduced colon length of the inflamed colon tissue (Fig. 2C). Furthermore, oral administration of luteolin, wedelolactone or apigenin alone also had a significant suppressive effect on the *in vivo* secretion of TNF-α from the inflamed colon tissues tested (Fig. 2D). Together, these data suggest that these phytochemicals, in particular luteolin, can play an important role in WCHA-suppressed colon inflammation.

### Luteolin and wedelolactone suppression of expression of IL17A (Th17) pathway-related and inflammasome-related genes

**Figure 3:**
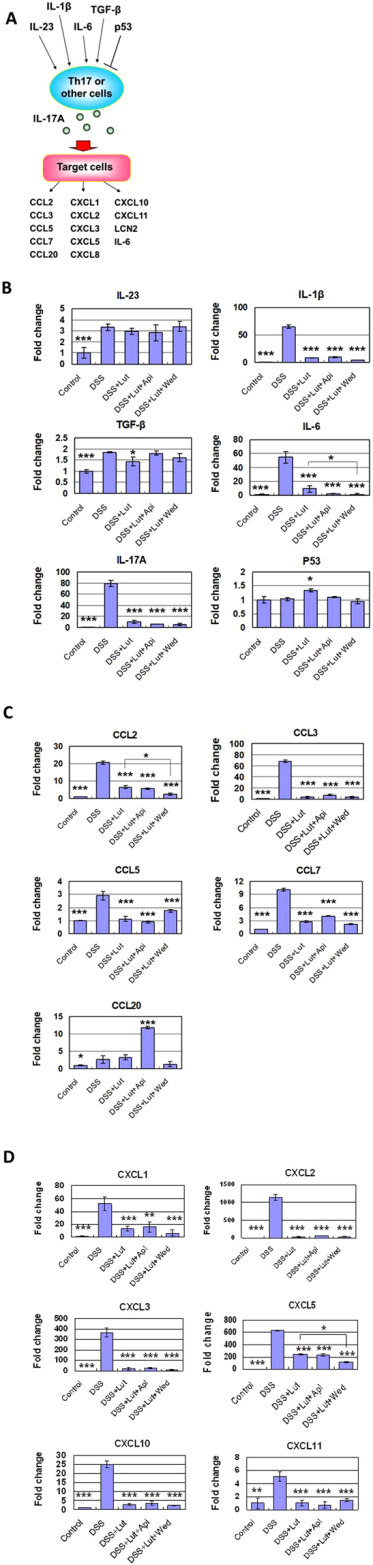
Suppressive effect of *LAW* on expression of IL17A (Th17) pathway-related genes. Next-generation sequencing (e.g., RNA-seq) approach was employed for analyzing the transcriptome of colorectal cells/tissues in DSS-induced colitis mice with/without treatment of specific phytochemicals (*LAW*). (**A**) The illustration of various upstream and downstream mediators in the IL-17A pathway. (**B**) Change in the expression level of upstream IL-17A pathway-related cytokine genes, including IL-23, IL-1β, TGF-β, IL-6, IL-17A and p53. Change in the expression level of downstream IL-17A pathway-related chemokine genes, including CCL2, CCL3, CCL5, CCL7, CCR20 (**C)**, CXCL1, CXCL2, CXCL3, CXCL5, CXCL10 and CXCL11 **(D**). Gene expression levels relative to those in the control group are represented by relative quantification values (fold changes). *P < 0.05, **P < 0.01, ***P < 0.001, significant change compared with the DSS-treated group.

To further investigate the pharmacological/protective mechanism of the anti-colitis activity of luteolin, the next-generation sequencing (e.g., RNA-seq) approach was employed to analyze the transcriptome of colorectal cells/tissues in DSS colitis mice with/without treatment with specific phytochemicals. Some DSS-treated mice were treated with luteolin only, others were treated with luteolin and apiginin, and yet others were co-treated with luteolin and wedelolactone, with all test phytochemicals at 0.5 mg/kg body weight. The expression of both upstream and downstream IL-17A pathway-related genes (Fig. 3A) in DSS-treated mice was found to be drastically suppressed by luteolin treatment (Fig. 3B–D). Luteolin treatment significantly suppressed the expression of many upstream activators in the IL-17 pathway, including IL-1β, IL-6 and IL-17A; the expression of these upstream activators had been drastically increased by DSS treatment (Fig. 3B). Interestingly, the expression activity of p53 was significantly increased by luteolin treatment. This result is also supportive for the controlling activity of luteolin in DSS-induced inflamed colon tissue. The results of NGS analysis also indicated that co-treatment of luteolin with wedelolactone can confer additive suppression on expression of other downstream components in the IL-17A pathway, including some of these IL-17A pathway-related cytokines or chemokines, including IL-6, CCL2 and CXCL5, as comparing with Luteolin treatment alone (Fig. 3B–D). These results together suggest that luteolin can effectively suppress the DSS-activated IL-17 pathway in inflamed colon tissue. Co-treatment with wedelolactone and luteolin may further suppress some of these gene expressions in an additive manner.

**Figure 4:**
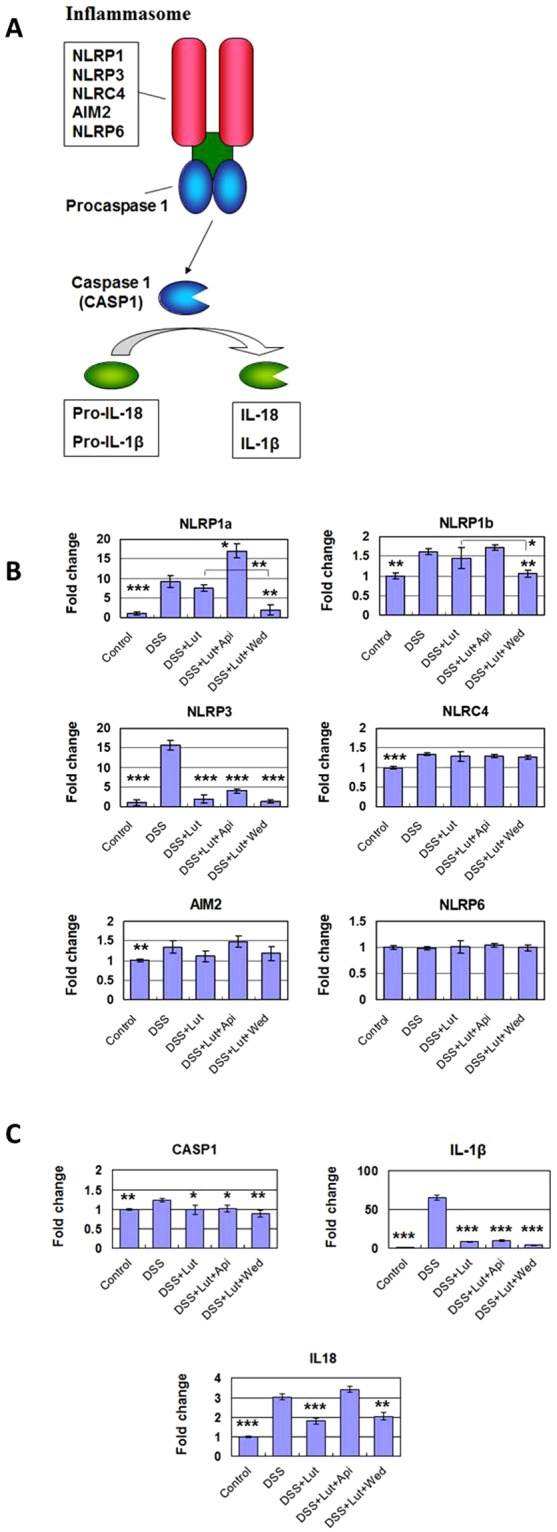
Luteolin and wedelolactone suppression of the expression of various inflammasome-related genes. NGS analysis was performed to determine the transcriptomic changes in colorectal cells/tissues of DSS-induced colitis mice with/without treatment with specific phytochemicals (LAW). (**A**) Illustration of the various upstream and downstream mediators in the inflammasome pathway. (**B**) Change in the expression of inflammasome component-related genes, including NLRP1a, NLRP1b, NLRP3, NLRC4, AIM2 and NLRP6, as a response to phytochemical treatments. (**C)** Change in the expression of various inflammasome pathway-related downstream genes, including CASP1, IL-1β and IL-18. Gene expression levels relative to those in the control group are represented by relative quantification values (fold changes). *P < 0.05, **P < 0.01, ***P < 0.001, significant change compared with the DSS-treated group.

Additional NGS analysis further indicated that luteolin and wedelolactone suppressed the expression of certain inflammasome-related genes (Fig. 4). For instance, luteolin treatment specifically suppressed the expression of NLRP3, which was drastically increased after DSS treatment (Fig. 4B). In addition, co-treatment with wedelolactone and luteolin further suppress the expression of NLRP1 (NLRP1a and NLRP1b) in DSS-treated colon tissues, as comparing with Luteolin treatment alone (Fig. 4B). Luteolin treatment also suppressed the expression of other downstream components of the inflammasome pathway, in particular, the DSS-induced IL-1β expression (Fig. 4C). These results suggest that treatment with luteolin and wedelolactone can suppress the expression activity of specific inflammasome-related genes (Fig. 4B), in inflamed colon tissue.

### Luteolin suppression of NLRP3 expression and IL-17A secretion in DSS-induced inflamed colon tissues

**Figure 5:**
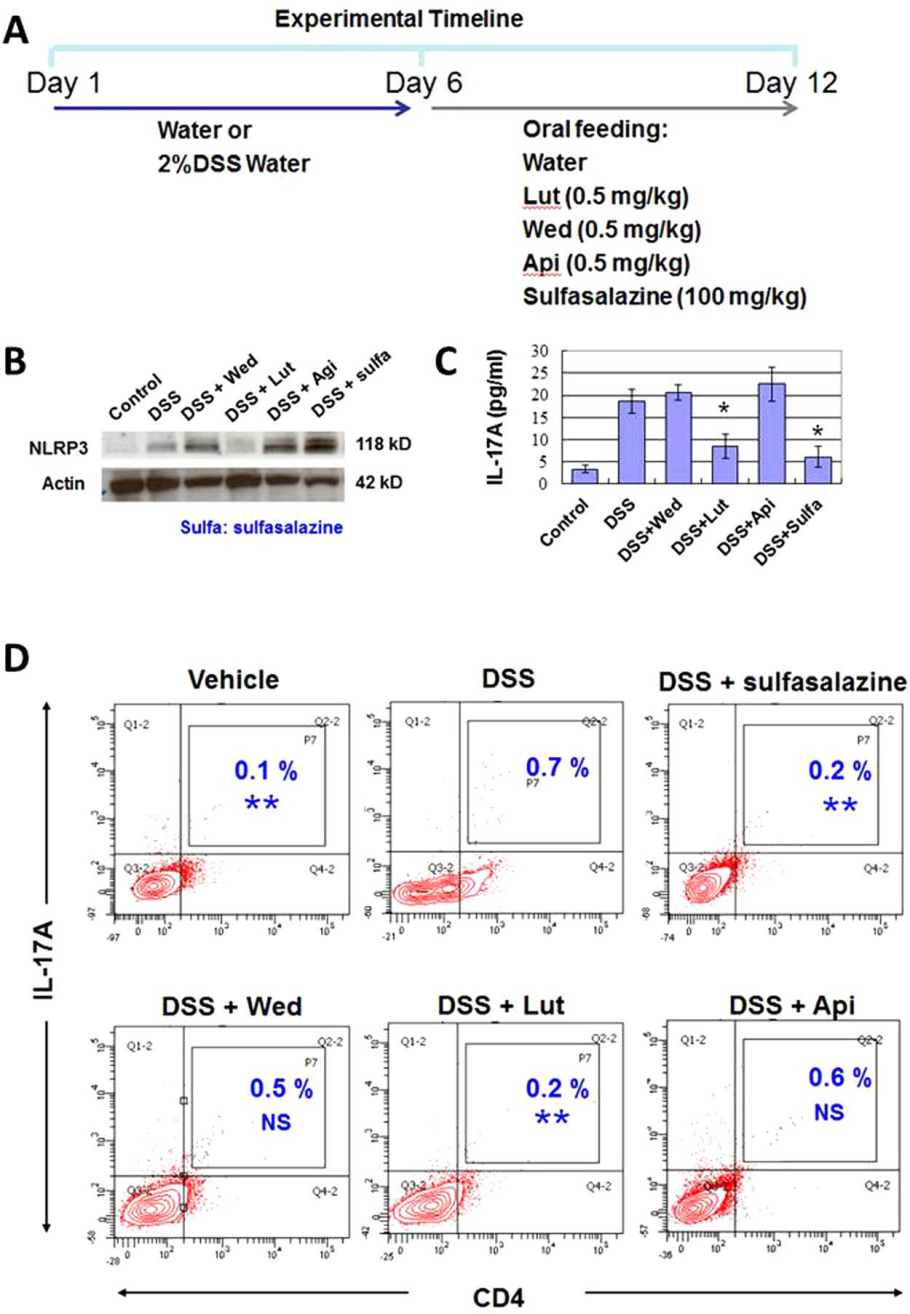
*In vivo* suppressive effect of luteolin on IL17A secretion and NLRP3 expression activities. (**A**) Experimental timeline for DSS-induced colitis and the subsequent oral feeding of different phytochemicals in test mice (**B**) Western blotting for expression of NLRP3 proteins, in response to *in vivo* treatment with LAW (0.5 mg/kg) or sulfasalazine (100 mg/kg) in mouse colon tissues. (**C**) ELISA for secretion of IL-17A in response to *in vivo* treatment with LAW or sulfasalazine. Effect on secretion of IL-17A for each phytochemical-treated group was compared with that of the DSS-treated group. (**D**) Effects of LAW or sulfasalazine on the population change in Th17 cells (CD4^+^IL-17A^+^) in colon tissues of test mice were quantified by flow cytometry analysis, and were compared with that of the DSS-treated group. Data are reported as the mean of 3 biological repeats. *P < 0.05; **P < 0.01; NS, not significant (2-tailed t-test).

To prove the suppressive effect of luteolin or wedelolactone on NLRP3 expression and IL-17A secretion *in vivo*, western blotting and ELISA were used to detect the expression and secretion activities of IL-17A and NLRP3 proteins, in response to *in vivo* treatment with luteolin, wedelolactone or sulfasalazine in mouse colon tissues. The experimental timeline (Fig. 5A) was designed for DSS-induced colitis and the subsequent oral feeding of different phytochemicals in test mice. Luteolin was found to significantly suppress the expression of NLRP3 in DSS-induced inflamed colon tissues (Fig. 5B). In addition, ELISA was employed to compare the secretion activity of IL-17A in response to *in vivo* treatment with luteolin, wedelolactone or sulfasalazine (Fig. 5C). The result was consistent with the result obtained from NGS analyses (Fig. 3B), indicating a strong suppressive effect of luteolin on IL17A secretion. The population of Th17 cells in colon tissue was also found to be decreased in respond to the administration of luteolin (Fig. 5D). Compared to the effects of sulfasalazine, a drug clinically used for IBD treatment, we found that luteolin treatment efficiently suppressed both NLRP3 expression and IL-17A secretion. However, sulfasalazine treatment was shown to suppress IL-17A secretion only, and not NLRP3 expression, in DSS-induced inflamed colon tissues.

### Luteolin-suppressed NLRP3 expression is potentially mediated by IL-17A-secreted cells

**Figure 6:**
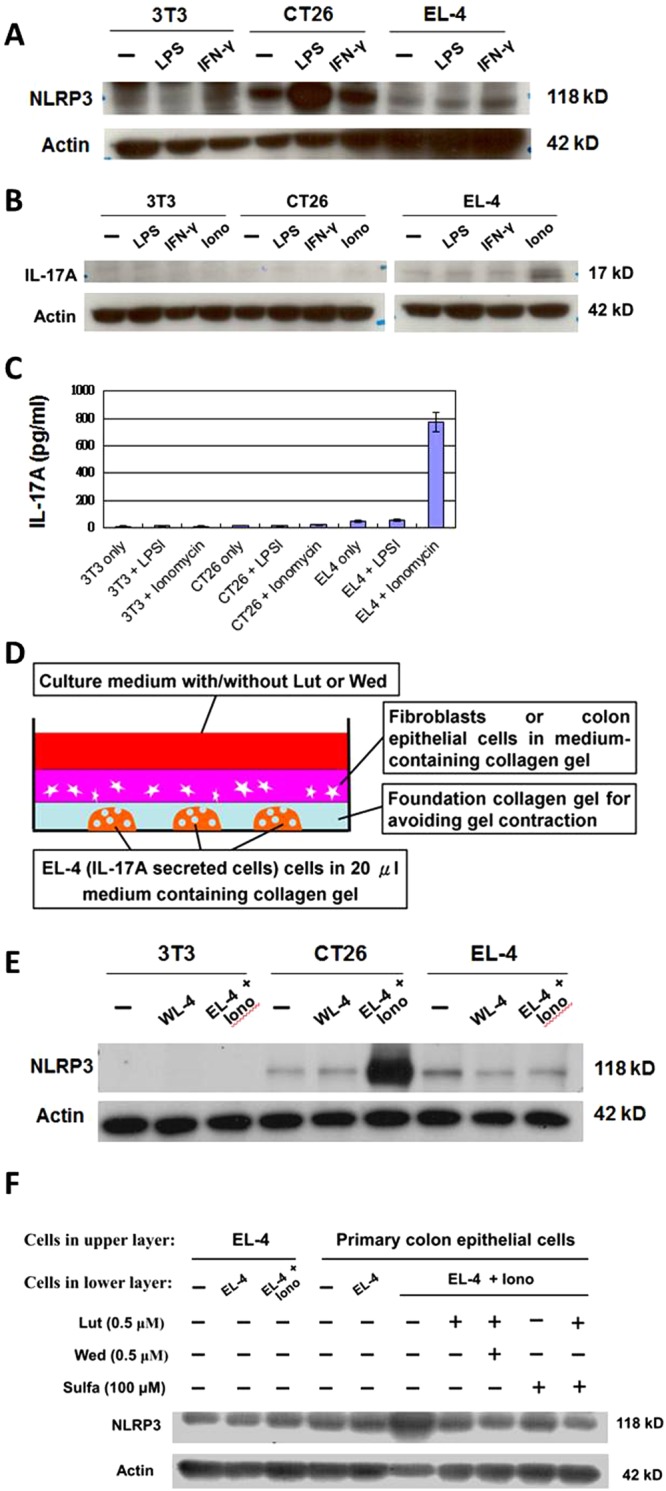
The IL-17A secretion in activating the formation of the NLRP3 inflammasome. (**A**) Western blot analyses. 3T3 (mouse fibroblasts), CT-24 (mouse colon carcinoma) or EL-4 (mouse lymphoma) cells were treated as indicated and compared for expression of NLRP3 and IL-17A. (**B**) Proteins, in response to the stimulation of LPS (0.5 μg/ml), IFN-γ (10 ng/ml) or ionomycin (1 μg/ml). Among various cell types, the expression level of IL-17A in EL-4 was inducible via ionomycin stimulation. (**C)** ELISA for secretion of IL-17A, in response to the stimulation of LPS, IFN-γ or ionomycin. The secretion level of IL-17A in EL-4 cells was inducible only via ionomycin stimulation. (**D**) Construction of a 3D cell co-culture system for studying the stimulation of IL-17A-secreted cells (EL-4) on the expression of specific inflammasome components. In this test, some CT-26 cells in the upper layer were co-cultured with EL-4 cells (as seeded in the lower layer), as indicated. (**E**) Western blot analyses of the expression of NLRP3 in mouse CT-26 cells, in response to the co-cultivation of EL-4 cells in a 3D cell co-culture system. (**F**) Western blot analyses of the expression of NLRP3 in primary colon epithelial cells, in response to the co-cultivation of control or ionomycin-treated EL-4 cells in a 3D cell co-culture system.

It is believed that the formation of the inflammasome complex can activate the secretion of some pro-inflammatory cytokines^7,8^, such as IL-1β, which is also the upstream activator for the IL-17A pathway. However, IL-17A also can induce the expression of NLRP3 at the post-transcriptional level^19^. These findings may together suggest that a positive feedback mechanism could play an important role between the formation of the NLRP3 inflammasome and activation of IL-17A signaling in the inflamed colon tissue of our tested mice. According to the data obtained from our current NGS analysis, the increase in IL-17A expression was observed of a much earlier phrase (48 h) than that for increase in NLRP3 or NLRP1 expression (96 h), after DSS treatment for 6 days. Therefore, we hypothesized here that the IL-17A secretion from IL-17A may act as an upstream activator for NLRP3 inflammasome formation in test target cells. To report the expression changes of IL-17A and NLRP3 inflammasome, expression levels of NLRP3 in 3T3 (mouse fibroblasts), CT-26 (mouse colon carcinoma) and EL4 (mouse lymphoma) cells were comparatively tested to that from the stimulation of LPS, IFN-γor ionomycin (Fig. 6A). Among these cell types, expression level of NLRP3 in CT-26, rather than in 3T3 or EL4 cells, was inducible via LPS stimulation (Fig. 6A). Therefore, CT26 cells were further used as reporter cells for NLRP3 expression in subsequent follow-up studies. Moreover, EL4 cells were used as IL-17A secreted cells, because the expression/secretion activity of IL-17A in EL4 is inducible via ionomycin (1 μg/ml) stimulation (Fig. 6B,C). Together, the EL4 cells and CT-26 cells therefore were used as IL-17A secreted cells and reporter cells for NLRP3 expression, respectively.

To further mimic the effect of Luteolin and Wedelolactone on cell-cell communication between IL-17A-secretted cells and NLRP3-expressed cells in tissue microenvironment, a 3D cell co-culture system was constructed to investigate the suppressive effect of phytochemicals on NLRP3 expression in presence of IL-17A-secreted cells (Fig. 6D). In this construction, the EL4 cells were grown in the lower layer. In the upper layer, the CT-26 cells (mouse colon carcinoma) were grown in medium-containing collagen gel. With this construction, western blot analyses were performed to compare the expression level of NLRP3 in mouse CT-26 cells, in response to the co-cultivation of EL4 cells. By comparison, the ionomycin-treated EL4 cells (IL-17A-secreted cells) were shown to specifically activate the expression of NLRP3 in CT-26 cells (Fig. 6E). This result suggests that the EL4 cells-secreted IL-17A may play an important role in activating the formation of the NLRP3 inflammasome. In some groups, 3T3 (mouse fibroblasts) or EL4 cells were also grown in the upper layer. However, the co-cultivation of EL4 cells in the lower layer did not activate the expression level of NLRP3 in 3T3 or EL4 cells in the upper layer (Fig. 6E). This result suggests that this increased NLRP3 expression is cell type-specific.

Using the same cell co-culture system, EL4 cells grown in the lower layer were also co-cultured with the mouse colon epithelial cells in the upper layer. EL4 cells pretreated with ionomycin were used to mimic the IL-17A secreted cells. With this construction, we could mimic and evaluate the effect of luteolin and wedelolactone on NLRP3 expression in primary colon epithelial cells in presence of IL-17A-secreted cells. The ionomycin-treated EL4 cells were found to significantly increase the level of NLRP3 expression in mouse colon epithelial cells (Fig. 6F). This result suggests that the expression of NLRP3 in colon epithelial cells is responsive to the activation of IL-17A-secreted cells. In addition, luteolin treatment (0.5 μM) was found to significantly suppress this increased NLRP3 expression in primary colon epithelial cells *in vitro* (Fig. 6F). This result suggests that luteolin can suppress NLRP3 expression under a pro-inflammatory condition. Although wedelolactone alone did not significantly suppress the expression level of NLRP3, which was drastically increased in DSS-treated colon tissue (Fig. 5B), co-treatment with wedelolactone and luteolin did cause additive suppression of the expression level of NLRP3 in colon epithelial cells, which were also co-cultivated with IL-17A-secreted EL4 cells (Fig. 6F). No cytotoxicity was detectable for luteolin or wedelolactone treatment at the employed concentration (Supplementary Fig. 1). This result suggests that wedelolactone may confer an additive suppression of NLRP3 expression in an indirect way.

### Alternative splicing activities of some lipid metabolism-related genes were regulated in luteolin-mediated anti-colitis

**Figure 7:**
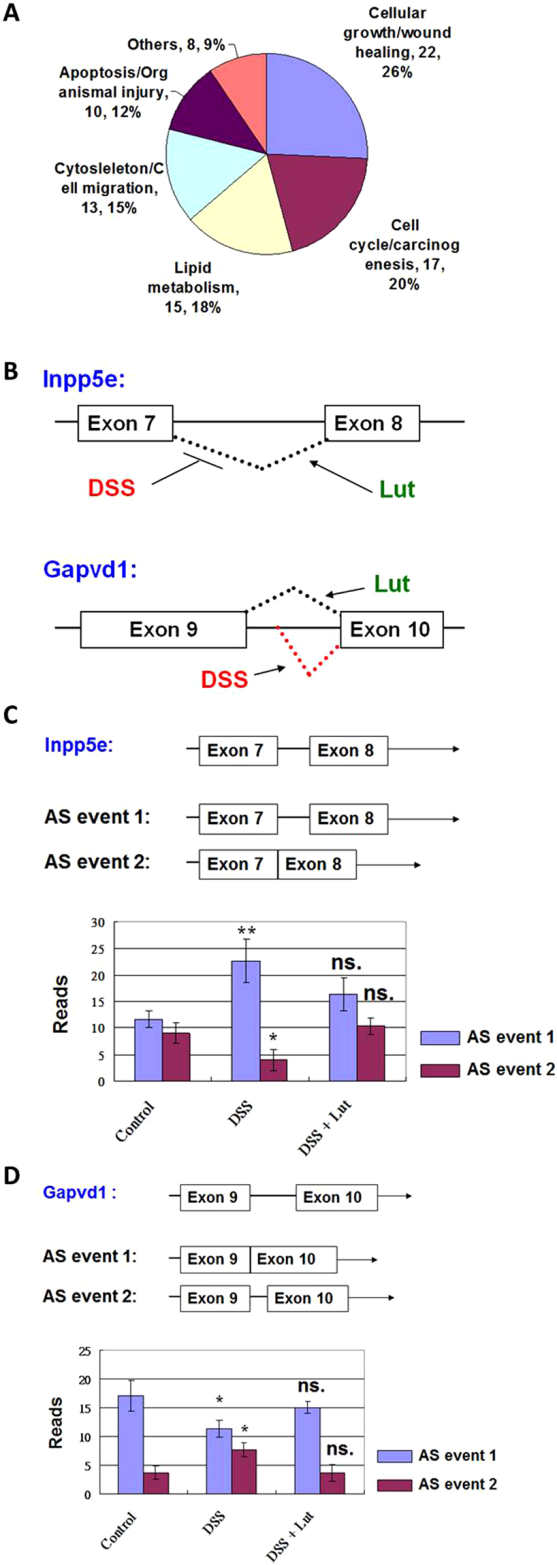
(**A**) DSS-induced alternative splicing. (**B**) Lipid synthesis-related genes. Changes in alternative splicing (AS) activity in response to luteolin treatment in DSS-induced inflamed colon tissues. The profiles of responsive AS events in DSS or phytochemical stimulations were systematically analyzed using MISO software. (**A**) DSS-induced AS activities. The changes in AS activities of many cellular growth/wound healing-related genes (26%), cell cycle/carcinogenesis-related genes (20%), lipid metabolism-related genes (18%), cytoskeleton/cell migration-related genes (15%) and apoptosis/organismal genes (12%) in DSS-induced inflamed colon tissue were evaluated using Ingenuity software. (**B**) The luteolin-mediated for AS action of some lipid synthesis-related genes, including Inpp5e and Gapvid1, were significantly disordered in the inflamed colon tissue. (**C**) For Inpp5e transcripts, the splicing activity of the intron between exons 7 and 8 was significantly suppressed by DSS treatment. However, luteolin treatment prevented this suppression. (**D**) For Gapvid1 transcripts, the splicing activity of the intron between exons 9 and 10 was also suppressed in the DSS-treated colon tissue. However, the luteolin treatment could prevent such suppression. Data are reported as mean ± SD (n = 3). *P < 0.05; **P < 0.01; NS, not significant (2-tailed t-test), significant difference compared with the control group (without DSS treatment).

Using NGS analysis, we also systematically analyzed the changes in alternative splicing (AS) activities for the whole transcriptome of the inflamed colon tissue. We systematically analyzed the splicing activities of each gene transcript using MISO (version 0.5.2) software. The profiles of responsive AS events for DSS or phytochemical stimulations were further investigated using Ingenuity software. By clustering analysis of gene function with significant change (s) in AS events in response to DSS treatment, the AS activities of many cellular growth/wound healing-related genes (26%), cell cycle/carcinogenesis-related genes (20%), lipid metabolism-related genes (18%), cytoskeleton/cell migration-related genes (15%) and apoptosis/organismal genes (12%) were regulated in DSS-induced inflamed colon tissue (Fig. 7A). Among these AS events, luteolin treatment was found to suppress specific AS activities of some lipid synthesis-related genes, including Inpp5e and Gapvid1, which were significantly changed in the inflamed colon tissue (Fig. 7B). To be more specific, the splicing activity of the intron between exons 7 and 8 in Inpp5e transcripts was significantly suppressed by DSS treatment. However, oral administration of luteolin could prevent this suppression (Fig. 7C). This result indicates that luteolin can maintain this splicing activity for expression of Inpp5e isoforms. The splicing activity of the intron between exons 9 and 10 in Gapvid1 transcripts was also suppressed in DSS-treated colon tissue. However, luteolin treatment could prevent this suppression (Fig. 7D). These results together suggest that disorders in the AS activities of some lipid metabolism-related genes also mediate the inflammatory response in colon tissue. Luteolin treatment can confer a controlling effect on these disorders at the post-transcriptional level.

## Discussion

**Figure 8:**
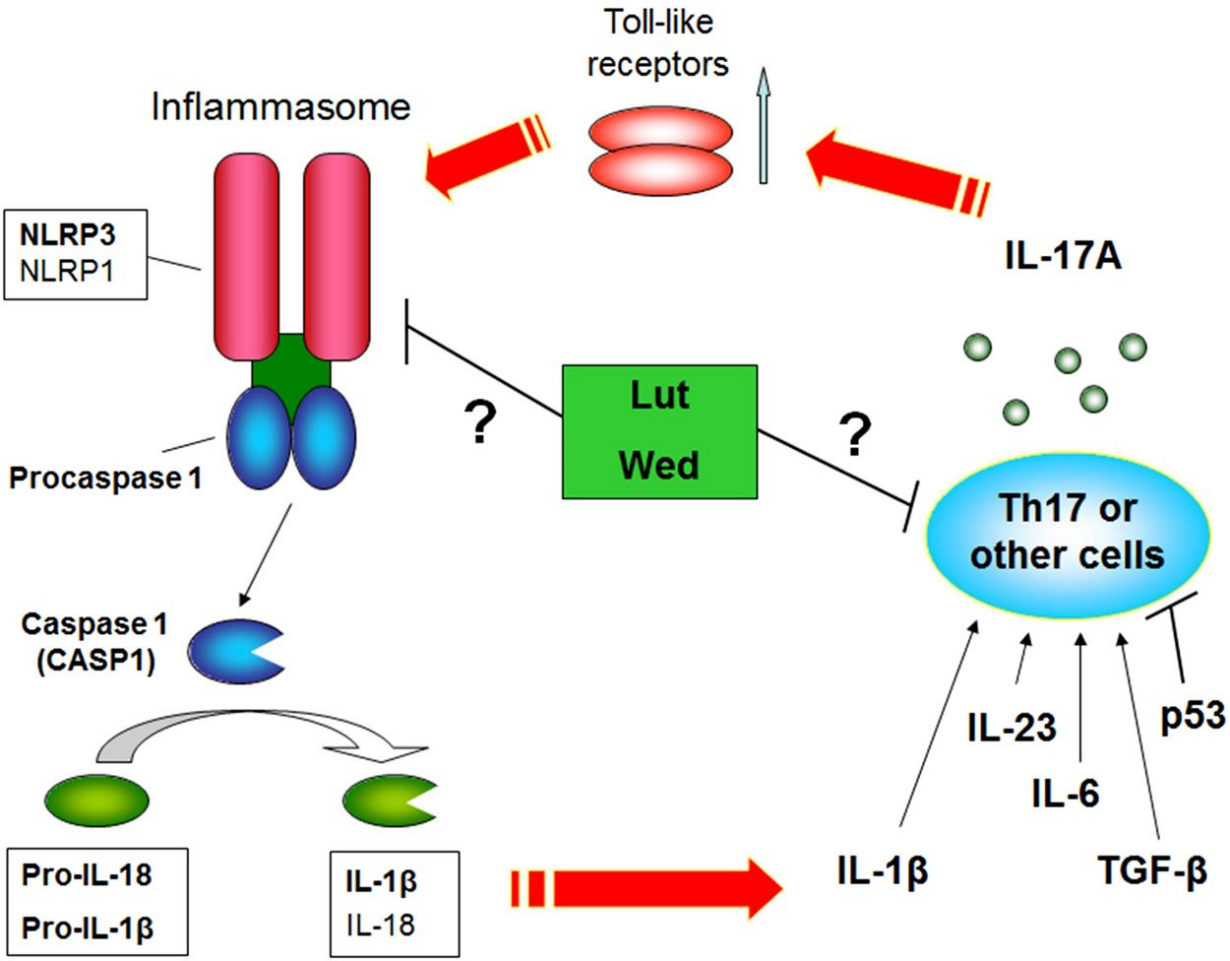
The proposed positive feedback mechanism for Th17 cell-secreted IL-17A and NLRP3 inflammasome activity in a circuit. In this proposed mechanism, specific natural compounds, such as luteolin, and wedelolactone, exhibited anti-colitis activity and suppressed IL-17A expression in Th17 cells and IL-17R-activated signaling at the post-transcriptional level, which further activated the specific TLR pathway and NLRP3 inflammasome. We also included the possibility that these phytochemicals can suppress the expression of specific inflammasome components, such as NLRP3 and NLRP1, via yet unknown mechanisms.

Through a consideration of the responsive genes for inflammatory stimulation (DSS) and specific phytochemical stimulations, a working hypothesis was proposed in this study for IL-17A secretion and NLRP3 inflammasome activity, which was observed as largely expressed in test colon epithelial cells, in a circuit (Fig. 8). In this proposed mechanism, the formation of the inflammasome complex can activate the secretion of some pro-inflammatory cytokines, such as IL-1β, which is also the upstream activator for the IL-17A pathway. Furthermore, IL-17A secretion from Th17 cells may also be the upstream activator for inflammasome formation in target cells, because it is known that IL-17A can activate inflammasome formation through the co-activation of a specific toll-like receptor pathway^12,13^. Luteolin or wedelolactone may exhibit anti-colitis activity through suppression of this positive feedback mechanism, via some specific targeting mechanisms. In addition, because other cell types, such as NK and innate lymphoid cells, also confer the IL-17A secretion activity^3^, whether these phytochemicals-mediated IL-17A suppression was via the modulation of Th17 cells is worth to be further studied.

With NGS analysis, we can not only obtain a profile of gene expression, but also systematically analyze the changes in alternative splicing/isoform activities in the whole transcriptome. If we had only considered the expression level of many lipid synthesis-related genes in this study, including Inpp5e and Gapvid1, we would not have detected the significant change at the gene expression level. But through alternative splicing analysis, we could gain more information at the posttranscriptional level. Consistent with our findings, previous studies also have reported the regulatory effect of different phytochemicals on AS activities of specific genes^20^. For example, the flavone apigenin was found to increase COX-2 mRNA stability via its specific effect on some RNA binding proteins, including TIAR and HuR, which could stabilize COX-2 transcripts^21^. Since the structure of luteolin is similar to that of apigenin, we also considered the possibility that luteolin can regulate the AS activities of specific lipid synthesis-related gene transcripts via some RNA binding proteins.

The luteolin, wedelolactone and salicylic acid-treated WC plant extract, exhibiting high and specific anti-inflammatory activities, may be useful in healthcare applications. The secondary metabolite profiles of high-level bio-active phytocompounds generate candidate botanical drug leads. Hence, we believe our findings may support the development of “medical food” and “food supplement” products for control/treatment of human IBD. In the future, more basic research knowledge can be expected from our experiments in identifying the exact or detailed biochemical processes and regulatory pathways (e.g., IL-17A pathway) involved in specific inflammasome activities formation, which may also dominate the anti-colitis activities of other medicinal herbs.

## Methods

### Compounds and preparation of *W*. *chinensis* extracts

*W*. *chinensis* was routinely collected from a Chinese medicinal herb store/farmer in Taipei City, Taiwan. The morphology, anatomy and genome sequence features of experimental plant materials were validated macroscopically, as previously reported^18,22,23^. Briefly, fresh WC plant material was air-dried and cut to 1 cm in size, then the material was subjected to 2 types of extraction methods, namely 95% ethanol extraction (WCE) and hot water extraction (WCH), and then concentrated by rotary evaporator. One part of the obtained crude extract was subjected to acid hydrolysis by HCl, pH 2, at 80 °C for 1 hour, neutralized by NaOH to pH 7, and then subjected to a desalting process. Crude extracts for both preparations (95% EtOH and hot water) with or without acid hydrolysis were then loaded into a C18 gel column and subjected to Flash liquid chromatography. The active fractions were identified by bioactivity-guided assays and pooled. Hence, 4 types of WC extracts were obtained, namely:

1. Hot water extract, denoted as WCH.
2. Hot water extract with acid hydrolysis, denoted as WCHA.
3. 95% EtOH extract, denoted as WCE.
4. 95% EtOH extract with acid hydrolysis, denoted as WCEA.

Using reversed phase C18 column chromatography (Biotage SNAP 120 KP-C18-HS Column), 4 major fractions, namely WCHA-F1, WCHA-F2, WCHA-F3 and WCHA-F4, were separated and collected from a 20~50% ethanol gradient. Since the WCHA-F3 fraction exhibited the highest anti-colitis activity in reversing the DSS-reduced colon length of the WCHA fractions tested, we further separated WCHA-F3 into 3 sub-fractions (WCHA-f1, -f2 and f3) by HPLC, in which ddH2O was a gradient eluted with acetonitrile (ACN) in a C18 column (COSMOSIL 5C18-AR-II 20 × 250 mm). The enriched active fraction was dried, frozen and stored at −80 °C until use. Pure compounds of luteolin (CAS: 491-70-3), wedelolactone (CAS: 524-12-9) and apigenin (CAS: 520-36-5) were purchased from Sigma (Sigma, St. Louis, MO).

### Mice

Seven- to 8-week-old male C57BL/6 mice were purchased from the National Laboratory Animal Center (Tainan, Taiwan). Five mice per group were fostered in 1 cage in a special pathogen-free (SPF) animal room kept at 22 °C with 55% relative humidity on a 14-h light/10-h dark cycle. The mice were fed a sterilized diet (Laboratory Autoclavable Rodent Diet 5010, USA) during their 1-week acclimatization.

### Dextran sulfate sodium (DSS)-induced acute murine colitis model

To evaluate possible key bio-active components for anti-colitis activity present in WCHA, colitis in C57 mice was induced in different test groups (6 mice/group) by feeding a 2% (w/v) DSS (molecular weight: 36,000–50,000 Da, MP Biochemicals, Solon) solution in drinking water from Day 0 to 6 (Fig. 1A), as described previously^17,18^. The vehicle control group was given regular drinking water for the entire experimental period. The DSS solution was filtered and changed every 3 days, and the mean DSS consumption was recorded. Throughout the experimental period (i.e., from 6 days before DSS treatment to 6 days post-DSS treatment, with a total 12-day treatment duration), mice in the DSS group and treatment groups were orally fed 2% (w/v) DSS solution only, various WC extracts or different phytochemicals (luteolin, wedelolactone and apigenin). Shikonin (SK; Tokyo Chemical Industry, Japan) was orally administered as a negative control (10 mg/kg) for suppression of DSS-induced colon inflammation. Another positive control, namely sulfasalazine (Sigma-Aldrich), was administered at a dosage of 200 mg/kg after the sterilized drinking water was changed to a 2% DSS. Sulfasalazine solution was dissolved in 0.5% cellulose; the 0.5% cellulose was dissolved in saline and autoclaved, and then Tween 80 was added to 1%, before adding the sulfasalazine solution. On Day 12, at the end of the experiment, all test mice were weighed and sacrificed by cervical dislocation.

### Next-generation sequencing (NGS) analysis

NGS technology was used to analyze the transcriptomes of colorectal cells/tissues in DSS-induced colitis mice with/without treatment with specific phytochemicals, and RNA-seq analyses of colon tissues in colitis mice were performed to elucidate the molecular signaling/protective mechanisms of the anti-colitis activities of each test phytochemical (luteolin, wedelolactone and apigenin). The colon tissues were isolated and frozen in liquid nitrogen immediately after sacrifice. Each collected colon tissue (<0.1 g) was then dissolved in 1 ml Trizol (Invitrogen) and 1 ml Tissue Protein Extraction Reagent (Thermo), for extraction of total RNA and cellular protein, respectively. Then total RNA was extracted using a TRIzol reagent (Invitrogen) according to the manufacturer’s instructions, and resuspended in 100 μl of diethyl pyrocarbonate-treated (DEPC) water. The RNA quantities and qualities of each individual test tissues were analyzed by Nanodrop and BioAnalyzer II reagents (Agilent). If all 3 samples from the same litter passed quality control (i.e., RNA integrity number [RIN] > 8.0), 10 ug of total RNA from all test samples would be pooled to reach a final 30 ug of total RNA for sequencing.

For paired-end mRNA-seq library preparation, we used Illumina mRNA-seq kits as described previously^24^. Briefly, a total of 30 ug RNA was used for mRNA enrichment by oligo-dT beads, followed by cation-catalyzed fragmentation for 4 min at 94 °C. The mRNA fragments were then converted into double-stranded cDNA by random priming, followed by end repair. The fragmented cDNAs were then ligated to the paired-end adaptors and subjected to size selection. For each pooled RNA sample, 3 sizes of ∼400 bp, ∼500 bp, and ∼550 bp were selected for the ligated cDNA. The 3-gel purified cDNA libraries were then subjected to 15 cycles of polymerase chain reaction (PCR) amplification and purified by Ampure beads (Beckman Agencourt). The absolute concentrations of the libraries were determined by Qubit fluorometry (Invitrogen) and a BioAnalyzer High Sensitivity DNA Kit (Agilent). Each size-selected mRNA-seq library was loaded in 1 lane of flow cells and paired-end 2 × 120 nt sequencing was conducted on an Illumina Genome Analyzer IIx, with a total of 3 lanes of data per pooled transcriptome. Library preparation and Illumina sequencing was carried out by the High Throughput Sequencing Core Facility, Biodiversity Research Center, Academia Sinica, Taiwan.

For the analysis of paired-end sequences, we trimmed all the paired-end sequencing reads from both ends of each cDNA fragment to 90 bp to reduce sequencing errors. Paired-end sequencing reads were mapped to the genome with TopHat (version 1.2.0)^25,26^. Only those paired-end reads mapped to the genome without mismatch were used for subsequent analyses. To calculate the expression levels, unique fragments were assigned to individual genes first, for initial abundance estimation, and the multiple-hit fragments were then redistributed to those genes based on the relative abundances of uniquely mapped fragments. The total amount of mappable fragments increased 2% by including redistributed multiple-hit fragments^24^. The normalized expression levels of genes, measured in fragments per kilobase of exon per million fragments mapped (FPKMs), were calculated using Cufflinks (version 1.0.3)^27^. Total mappable fragments on each chromosome were calculated by SAMtools^28^.

### Alternative splicing analysis

MISO v0.5.2 was used for alternative splicing event analysis, difference detection and visualization. MISO (Mixture-of-Isoforms) is a probabilistic framework that quantitates the expression level of alternatively spliced genes from RNA-Seq data, and identifies differentially regulated isoforms or exons across samples. By modeling the generative process by which reads are produced from isoforms in RNA-Seq, the MISO model uses Bayesian inference to compute the probability that a read originated from a particular isoform. MISO treats the expression level of a set of isoforms as a random variable and estimates a distribution over the values of this variable^29^. The estimation algorithm is based on sampling, and falls into the family of techniques known as Markov Chain Monte Carlo (MCMC). Percent Spliced In (PSI) was used in the difference detection with Δψ bigger than 0.2 and Bayes factor >10.

### Cell lines

The 3T3 (mouse fibroblasts), CT-26 (mouse colon carcinoma) and EL4 (mouse lymphoma) cells were originally obtained from American Type Culture Collection (ATCC). 3T3 and EL4 cells were maintained in DMEM (Invitrogen, Carlsbad, CA) supplemented with 3.7 g/L sodium bicarbonate, 3.6 g/L HEPES and 10% FBS. The CT-26 cells were maintained in RPMI-1640 (Invitrogen, Carlsbad, CA) complete medium supplemented with 10% fetal bovine serum (FBS). All cells were grown in a 5% CO2 incubator at 37 °C. After the cells had grown to 50% confluence and were treated with test compounds, both adherent and floating 4T1 cells were scraped at 24 h or 48 h post-treatment with different test compounds and were centrifuged for 3 min (1,200 rpm). After removing the supernatant, the cells were resuspended in Mammalian Protein Extraction Reagent (Thermo) and frozen at −80 °C. Prior to use, cell lysates were thawed and centrifuged at 12,000 rpm for 30 min, and the protein concentrations were determined using the BCA assay (Pierce, Rockford, IL, USA).

### Western blot assay

Cell lysate samples were resolved by SDS PAGE using 10% or 12% stepwise gels. The resolved proteins were transferred onto a PVDF membrane (Novex, San Diego, CA) and blotted with anti-NLRP3 (rabbit polyclonal; Abcam), anti-IL-17A (rat monoclonal; Abcam), or anti-β-actin (rabbit polyclonal; Abcam). The membrane was blocked with 5% non-fat dry milk in PBST buffer [phosphate-buffered saline (PBS) containing 0.1% Tween 20] for 60 min at room temperature. Blotted membranes were then incubated overnight at 4 °C with specific, commercially available antibodies (1:1,000 dilutions). Loading of equal amounts of protein was assessed using mouse β-actin protein as a reference. The blots were rinsed 3 times with PBST buffer for 5 min each. Washed blots were incubated with HRP-conjugated secondary antibody (goat polyclonal; Abcam; 1:100,000 dilution) and washed again 3 times with PBST buffer. The transferred proteins were visualized with an enhanced chemiluminescence (ECL) detection kit (Amersham Pharmacia Biotech, Buckinghamshire).

### Flow cytometry assays

At the end of acute colitis experiment, freshly excised colon tissues from test mice were flushed with sterilized PBS to remove soiling. For detection of Th17 cells (CD4^+^IL-17A^+^), cells from mouse colon tissue in each group were collected and stained for 30 min at 4 °C with antibodies against specific cell markers, including FITC-conjugated anti-mouse CD4 and PE-conjugated anti-mouse IL-17A. All three antibodies were obtained from Biolegend, (San Diego, CA). The percentages of Th17 cells were gated on CD4^+^IL-17A^+^ cells. Flow cytometry was performed on a FACS LSR II (BD, Netherlands) machine at the Agricultural Biotechnology Research Center (ABRC) in Academia Sinica.

### 3D cell co-culture system

To mimic the effect of phytochemicals on cell-cell communication in a tissue microenvironment, a 3-dimensional (3D) co-culture system^30^ was employed for the maintenance of IL-17A-secreted cells and NLRP3-expressed cells in adjacent collagen gels. Briefly, rat-tail collagen solution dissolved in acetic acid was neutralized by 1 N NaOH and then mixed with 10 × PBS at 4 °C at a ratio of 9:1. To construct a solid IL-17A-secreted-cell mass, EL4 cells in each corresponding culture medium (1 × 10^6^ cells/ml) were mixed with 4 mg/ml collagen solution at a ratio of 1:1. In some groups, EL4 cells were treated by ionomycin (1 μg/ml) for induction of IL-17A secretion. Four drops of collagen-tumor cell mixture (1 × 10^4^ cells/10 μl/drop) were immediately and separately loaded on culture substratum in each well of a 6-well plate. The plates were then turned over and kept in a CO2 incubator at 37 °C for collagen gelation. To prepare the foundation collagen gel (Fig. 6D), culture media were mixed with 4 mg/ml collagen solution at a ratio of 1:1 (final concentration: 2 mg/ml). Aliquots of 1 ml of foundation collagen solution were loaded on a culture substratum and each was covered with a collagen-tumor cell mixture. The culture plates were then kept in a CO2 incubator at 37 °C for another 10 min. To prepare the fibroblast- or primary colon epithelial cells-containing collagen gel, 3T3 or mouse colon epithelial cells in each corresponding culture medium (3 × 10^6^ cells/ml) were mixed with 4 mg/ml collagen solution at a ratio of 3:1. 1 ml fibroblast- or epithelial cells-containing collagen solution (1 × 10^6^ cells/1 ml/well) was immediately loaded on each solid foundation collagen gel. Finally, culture media for cell type in the upper layer were added onto the top layer and were suspended with test phytochemicals for different compound treatments. After 24 or 48 h co-culture of EL4 cells with CT26 or epithelial cells, the upper layer of fibroblasts- or epithelial cells-containing collagen gel was separated from the foundation gel using a pipet tip to smoothly scrape the collagen layer. Each collected collagen gel was then minced in an Eppendorf tube using scissors and dissolved in 1 ml Tissue Protein Extraction Reagent (Thermo), for extraction of total cellular protein.

### ELISA

The secretion levels of mouse TNF-α and IL-17A in culture media were measured using an enzyme-linked immunosorbent assay (ELISA) kit (R&D systems, Minneapolis, MN), according to the manufacturer’s protocol. All assays were performed in triplicates.

### Statistical Analysis

Results are expressed as mean ± S.D. of a representative experiment performed in triplicates. Statistical analysis was performed using an unpaired, 2-tailed Student’s t-test (*P < 0.05; **P < 0.01; ***P < 0.001; n.s, no significance).

### Ethics approval

We confirm that the animal experiments were approved by Institutional Animal Care and Use Committee (IACUC) of Academia Sinica ethics committee. The reference number is 12-01-304. We also confirm that all methods were performed in accordance with the relevant guidelines and regulations by Academia Sinica ethics committee.

## Electronic supplementary material

Supplementary Figure 1
