# Supplementary material for: Transcriptomic analysis reveals a controlling mechanism for NLRP3 and IL-17A in dextran sulfate sodium (DSS)-induced colitis
Source: Sci Rep. 2018 Oct 8;8:14927. doi: 10.1038/s41598-018-33204-5 (PMC6175949; doi:10.1038/s41598-018-33204-5)

**Transcriptomic analysis reveals a controlling mechanism for NLRP3 and IL-17A in dextran sulfate sodium (DSS)-induced colitis**

Tien-Jen Lin^1, 2, 3*^,Shu-Yi Yin^4^, Pei-Wen Hsiao^4^, Ning-Sun Yang^4^, and I-Jen Wang^5,6,7^*

**Supplementary Figure 1.**

**MTT assays:**


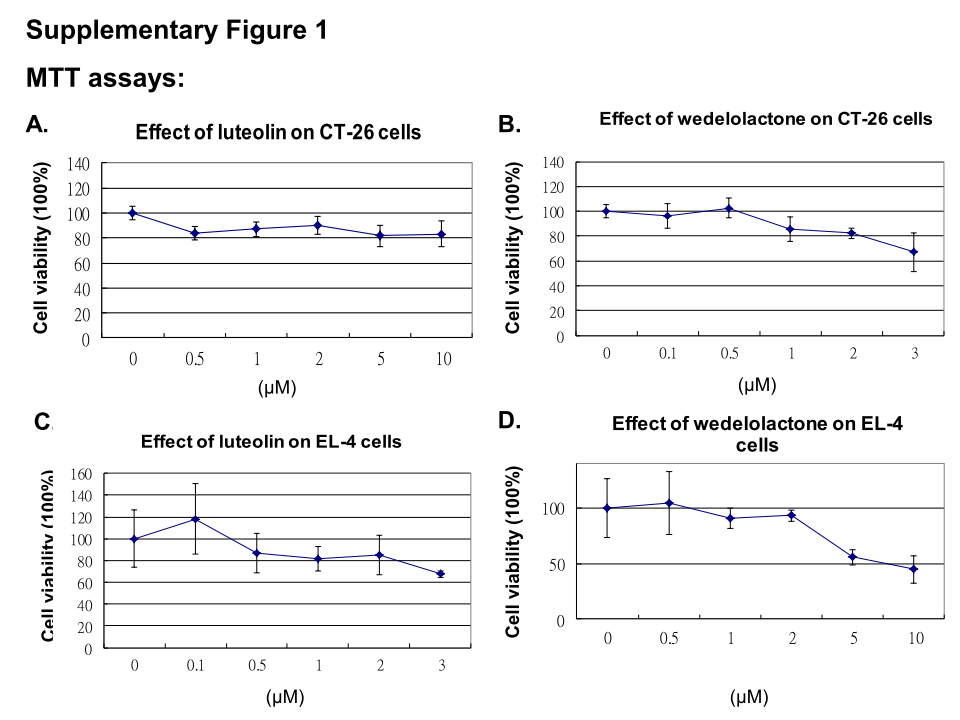

Supplement: Supplementary file 1 — Supplementary Figure 1 [file 41598_2018_33204_MOESM1_ESM.docx]
